# Breaking the paradigm: early insights from mammalian DNA *breakomes*

**DOI:** 10.1111/febs.15849

**Published:** 2021-05-01

**Authors:** Xanita Saayman, Fumiko Esashi

**Affiliations:** ^1^ Sir William Dunn School of Pathology University of Oxford UK

**Keywords:** DNA breaks, genome instability, next‐generation sequencing, replication, transcription

## Abstract

DNA double‐strand breaks (DSBs) can result from both exogenous and endogenous sources and are potentially toxic lesions to the human genome. If improperly repaired, DSBs can threaten genome integrity and contribute to premature ageing, neurodegenerative disorders and carcinogenesis. Through decades of work on genome stability, it has become evident that certain regions of the genome are inherently more prone to breakage than others, known as genome instability hotspots. Recent advancements in sequencing‐based technologies now enable the profiling of genome‐wide distributions of DSBs, also known as *breakomes*, to systematically map these instability hotspots. Here, we review the application of these technologies and their implications for our current understanding of the genomic regions most likely to drive genome instability. These breakomes ultimately highlight both new and established breakage hotspots including actively transcribed regions, loop boundaries and early‐replicating regions of the genome. Further, these breakomes challenge the paradigm that DNA breakage primarily occurs in hard‐to‐replicate regions. With these advancements, we begin to gain insights into the biological mechanisms both invoking and protecting against genome instability.

## Graphical abstract

Recent advancements in sequencing‐based technologies allow the detection of DNA breaks genome‐wide, providing an unbiased snapshot of genome‐wide break susceptibility in various experimental conditions. Pairing these data with genomic and epigenomic information, researchers are beginning to gain insight into the molecular mechanisms both provoking and protecting against DNA breaks. Here, we review the application of these technologies and their implications for our current understanding of the drivers of genome instability.

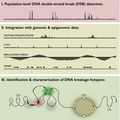

**Abbreviations**
CFS: Common fragile site
CTCF: CCCTC‐binding factor
DSB: Double‐strand break
ERFS: Early‐replicating fragile site
FRA16D: Fragile site, aphidicolin type, common, fra(16)(q23.2)
FRA3B: Fragile site, aphidicolin type, common, fra(3)(p14.2)
MCF7: Michigan Cancer Foundation‐7 (breast cancer cell line)
NHEK: Normal human epidermal keratinocytes
RAD51: DNA repair protein RAD51 homolog 1
rDNA: Ribosomal DNA
RPA: Replication protein A
SSB: Single‐strand break
TAD: Topologically associated domain
TOP I: Topoisomerase I
TOP II: Topoisomerase II

## Introduction

The accurate preservation and faithful transmission of genetic information is arguably the single most important task of the living organism. This is no small feat, given the considerable range and extent of DNA‐damaging threats experienced by any given cell. It has been estimated that human cells undergo ~ 70 000 lesions per day, of which 25–50 of these are double‐strand breaks (DSBs) [1, 2]. If improperly repaired, DSBs can result in both base mutations and chromosomal rearrangements, such as inversions, amplifications, deletions and translocations. Ultimately, these events can underlie a range of pathologies related to genome instability including carcinogenesis and premature ageing [3, 4, 5].

Double‐strand breaks in the human genome can result from extrinsic sources such as ionizing or ultraviolet radiation as well as intrinsic sources such as replication, transcription and chromatin looping [6, 7, 8, 9, 10]. While DSBs from extrinsic sources tend to be more randomly distributed across the genome, DSBs from intrinsic sources tend to reoccur at specific genome locations. This nonrandom distribution indicates that there are regions of the genome that are unusually susceptible to DSB formation, also known as genome instability hotspots.

Historically, the mapping of genome instability hotspots has been conducted upon exposure to exogenous genotoxic stress (e.g. drug‐induced replication perturbation, topoisomerase inhibitors) or genetic perturbation of DNA metabolism (e.g. mutation of the replication machinery), thereby increasing the incidence of DNA breakage and enabling the detection of DSBs. From these works, a set of genome instability hotspots have been defined, including regions of low origin density, DNA repeats, common or rare fragile sites, slow replicating zones, non‐B DNA structures and telomeres [11, 12, 13, 14, 15, 16, 17], reviewed in Ref. [18]. However, given that these instability hotspots were typically characterized following exogenous stress or in mutant genotypes, it remains unclear whether these regions accurately represent genome instability hotspots in unperturbed cell populations.

With recent developments in sequencing‐based technologies, we are now beginning to gain the sensitivity to quantitatively and directly detect physical DNA breaks in populations of cells. Mapping genome‐wide break profiles, also known as DNA *breakomes*, identifies inherent DNA break susceptibility across the genome. Here, we review the findings following the application of these recently developed technologies profiling DNA breakomes in various experimental conditions (Table 1), and what is known of the mechanisms underlying DNA break susceptibility at the identified instability hotspots. This review focuses on the biological implications of breakome studies rather than a systematic comparison of the different technologies available, which has been reviewed extensively elsewhere [19]. Overviewing these emerging insights, we also aim to discuss how DNA breakomes challenge current paradigms of genome instability hotspots.

**Table 1:** Mapping genome instability hotspots genome‐wide. A tabulation of recently published genome‐wide DNA breakomes identifying genome instability hotspots. The method applied, biological conditions assessed and mammalian organism used are specified for each reference. On the far right, it is noted whether the respective study identified an enrichment of DNA breaks at common genome instability hotspots such as actively transcribed regions, insulating regions, early‐replicating regions, fragile sites, centromeres or telomeres. BLESS, breaks labelling, enrichment of streptavidin and next‐generation sequencing; BLISS, breaks labelling *in situ* and sequencing; DSBCapture, double‐strand DNA break capture; END‐seq, end sequencing; RAFT‐seq, rapid amplification of form termini sequencing; sBLISS, suspension‐cell breaks labelling *in situ* and sequencing.

Reference	Method	Condition	Organism	Actively transcribed regions	Loop boundaries	Early‐replicating regions	Fragile sites	(Peri)centromeres / telomeres
Crosetto *et al*. (2013) [23]	BLESS	Replication stress (APH)	*M. musculus, H. sapiens*	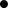			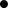	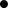
Baranello *et al*. (2014) [20]	SSB‐seq / DSB‐seq	+/− Top II poison (etoposide)	*H. sapiens* (HCT116)	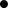				
Yang *et al*. (2015) [24]	BLESS	+/− Anthracyclines, Top II poison (etoposide)	*M. musculus* (MSCC‐CK1)	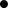				
Tchurikov *et al*. (2015)	RAFT‐seq		*H. sapiens* (HEK293T)	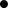				
Lensing *et al*. (2016) [27]	DSBCapture		*H. sapiens* (HeLa, U2OS, NHEK)	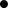	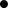			
Yan *et al*. (2017) [25]	BLISS	+/− Top II poison (etoposide)	*M. musculus* (primary liver cells), *H. sapiens* (U2OS, HEK293, KBM7)	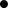				
Canela *et al*. (2017)	END‐seq	+/− Top II poison (etoposide)	*M. musculus* (activated B‐cells), *H. sapiens* (activated B‐cells)	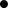	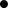			
Mourad *et al*. (2018)	DSBCapture, BLESS, BLISS, END‐seq		*H. sapiens* (NHEK, U2OS, KBM7, MCF7)	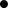	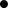			
Tubbs *et al*. (2018)	END‐seq	Low/high‐dose replication stress (HU)	*M. musculus* (primary splenic B‐cells), *H. sapiens* (HCT116)			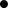	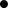	
Canela *et al*. (2019) [28]	END‐seq	Top II poison (etoposide)	MEFs, B‐cells, HCT116	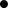	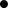			
Ballinger *et al*. (2019) [89]	DSBCapture, BLISS and BLESS		*H. sapiens* (MCF7, NHEK, K562)	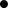	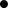	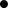		
Hazan *et al*. (2019) [30]	BLISS		*H. sapiens* MCF7, MCF10A, BJ, EndoC), *M. musculus* (embryonic, neural stem cells)	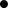	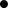			
Gothe *et al*. (2019) [26]	sBLISS	+/− Top II poison (etoposide)	*H. sapiens* (TK6, U2OS, K562)	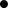	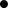			
Promonet *et al*. (2020) [58]	i‐BLESS	+/− Topoisomerase I (shRNA)	*H. sapiens* (HeLa)	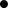				
Chakraborty *et al*. (2020) [80]	Break‐seq	+/− Replication stress (APH)	*H. sapiens* (lymphoblasts, wild‐type and fragile X)	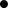				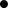

## Genome instability hotspots

**Fig. 1:**
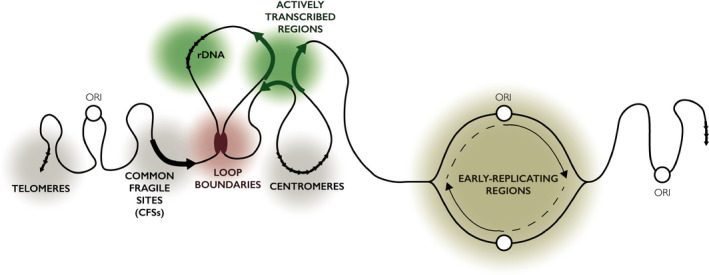
Overview of genome instability hotspots. DNA breakomes profile genome‐wide distributions of DNA DSBs to objectively identify genome instability hotspots in various experimental settings. In the absence of exogenous perturbation, genome instability hotspots most frequently occur at actively transcribed regions and rDNA (green), loop boundary sites between TADs (red), early‐replicating regions (yellow). Upon exogenously induced replication stress, genome instability hotspots expand to include hard‐to‐replicate regions such as CFSs, centromeres and telomeres (grey).

DNA breakomes conducted in the last decade have identified genome instability hotspots such as actively transcribed regions, loop boundaries, fragile sites, early‐replicating regions, telomeres and centromeres (Fig. 1). Certain instability hotspots (e.g. actively transcribed regions, loop boundaries and early‐replicating regions) appear to form DSBs innately, whereas others (e.g. common fragile sites (CFSs), telomeres and centromeres) become susceptible to breakage only in certain physiological settings. Here, we discuss each of these genome instability hotspots, the conditions in which they express their instability and how this corresponds to existing paradigms of genome instability throughout the genome.

### Actively transcribed regions: transcription as a source of genome instability

In recent years, it has become increasingly apparent that breakome landscapes are considerably skewed towards regions that are actively transcribed [10, 29]. Indeed, sites of active transcription are one of the only regions to be detected in breakomes using noncancerous mammalian cells without any exogenous perturbation [25, 27, 30]. Further, a direct correlation between the rates of active transcription and DSB formation has also been consistently reported [22, 24, 26, 27, 31, 32]. This trend remains apparent across a range of experimental conditions including replication stress and topoisomerase II (TOP II) poisoning, suggesting that transcription poses a threat to genome stability in various contexts [10, 20, 21, 23, 24, 25, 26, 28, 29]. These findings imply that genomic regions with high densities of transcriptionally active loci may become DNA instability hotspots. Alternatively, actively transcribed instability hotspots may not refer to consecutive sites in the one‐dimensional genome, but rather multiple independent sites brought together to form three‐dimensional compartments, such as in transcription hubs. In this sense, actively transcribed genome instability hotspots would refer to a position in nuclear space rather than a linear coordinate range within the human genome.

Increasing transcriptional activity acutely increases both mutation and recombination rates [9, 33, 34, 35, 36]. While this link between mutation rates, recombination and transcriptional activity has been recognized for several decades, the mechanistic details of transcription‐related DNA fragility are only now beginning to be revealed. Several hypotheses have been proposed to explain the inherent fragility of actively transcribed regions, which can be generally classified into replication‐dependent or replication‐independent mechanisms.

#### Replication‐dependent breaks

Transcription–replication collisions are rare in healthy human cells, as eukaryotes compartmentalize replication and transcription in distinct nuclear territories at distinct times [37, 38]. However, collisions do occasionally occur at exceptionally long (> 800 kbp) genes, or when replication programmes are perturbed using replication inhibitors [39]. Indeed, genes that are both late‐replicating and transcriptionally active have been identified as being susceptible to DNA instability, but only when the replication programme is perturbed [21, 39, 40, 41].

**Fig. 2:**
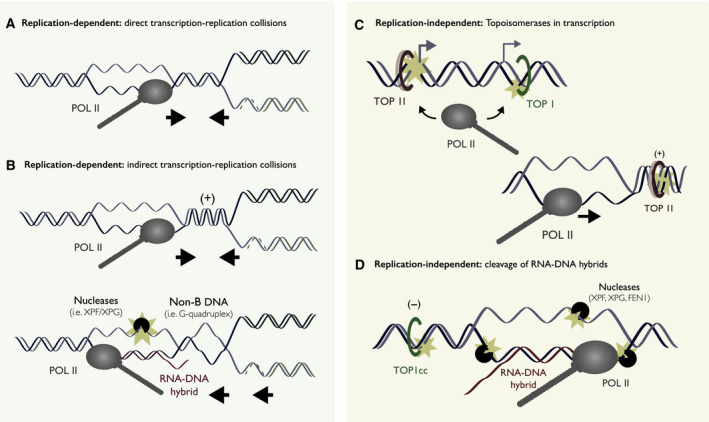
Active transcription as a source of genome instability. (A) During DNA replication, transcriptionally active regions are more prone to genome instability through direct transcription–replication collisions. (B) Indirect transcription–replication collisions are mediated by elements such as RNA‐DNA hybrids, non‐B DNA structures and DNA supercoiling. Transcription–replication collisions are typically observed when these elements are artificially stabilized or when the replication or transcription programmes are perturbed. (C) Independent of DNA replication, TOP I and TOP II can generate transient SSBs or DSBs in transcriptional activation. Both TOP I and TOP II promote expression of highly expressed genes and enhance the recruitment of RNA polymerase II. In addition, supercoiling behind and ahead of the transcription bubble are resolved by the combined actions of TOP I and TOP II to promote transcription. (D) Independent of DNA replication, transcription can result in DSBs through two single‐strand breaks (SSBs) generated on opposing DNA strands. Dual processing of topoisomerase I cleavage complexes (TOP1cc) and RNA‐DNA hybrids by endonucleases (i.e. XPF, XPG, FEN1) results in proximal SSBs, forming DSBs upon strand separation. Stabilization of RNA‐DNA hybrids is required for break formation.

Replication–transcription collisions can be induced either directly through physical obstructions of the respective machineries (Fig. 2A), or indirectly through other elements that impede replication fork progression (Fig. 2B). Direct head‐on replication–transcription collisions are more prone to breakage than codirectional collisions, although both are able to interfere with replication progression in eukaryotes [42, 43, 44, 45]. Indirect collisions can be mediated by noncanonical DNA structures formed during the normal process of replication or transcription, such as DNA supercoiling, non‐B DNA structures and RNA‐DNA hybrids [46, 47, 48, 49]. In either case, stalled replication forks can be cleaved by structure‐specific endonucleases to generate DSBs [8, 50].

Of the elements capable of impeding fork progression through indirect transcription–replication collisions, RNA‐DNA hybrids have received much interest in recent years as potential sources of genome instability. RNA‐DNA hybrids, or R‐loops, are formed co‐transcriptionally by hybridization of nascently transcribed RNA to the template DNA strand. As a result, they are enriched at active promoters and open chromatin, but are also frequently found at insulators, enhancers and terminators [51, 52]. While native RNA‐DNA hybrids are transient and appear to be mostly innocuous, genotypes that stabilize these structures (i.e. RNA processing and regulatory mutants) result in an accumulation of R‐loops that are commonly associated with DNA damage [53, 54, 55, 56]. These stabilized structures can then physically impede replication fork progression, resulting in a stalled fork or incomplete replication, but may also promote the formation of single‐stranded breaks (SSBs) or even DSBs by endonucleolytic cleavage on the exposed single‐stranded DNA [49, 57, 58, 59, 60]. More recently, however, the mechanism of toxicity induced by these structures has been called into question with evidence that R‐loop‐triggered chromatin compaction may be the true underlying cause of genome instability in situations with persistent RNA‐DNA hybrid accumulation [61].

#### Replication‐independent breaks

Transcription can also trigger DNA breaks independently of DNA replication, events that are frequently observed in postmitotic (nondividing) cell populations [62]. Replication‐independent DSBs within transcriptionally active regions are proposed to be mediated through at least two mechanisms: the action of topoisomerases in transcriptional initiation and elongation (Fig. 2C), and the induction of two proximal SSBs on opposite DNA strands at RNA‐DNA hybrids (Fig. 2D).

The first mechanism is catalysed by topoisomerase I (TOP I) and TOP II promoting gene expression (Fig. 2C) [63]. Topoisomerases are a class of enzymes that generate temporary DNA breaks to resolve topological constraints during replication and transcription, among many other functions [64, 65, 66, 67]. It is widely considered that topoisomerases (e.g. TOP IIβ) cleave DNA to relieve topological constraints hindering RNA polymerase progression, allowing transcription elongation of long genes [68]. However, there is emerging evidence that both TOP I and TOP II can also function in transcriptional initiation itself. Although the mechanistic details behind this role are not fully understood, it has been shown that both TOP I and TOP II can promote the initial recruitment of RNA polymerase II [69]. TOP I generates DNA nicks (SSBs) to initiate RNA synthesis at many enhancer sites [70]. TOP IIβ, on the other hand, activates transcription by introducing DSBs at the promoters of early‐response genes following a wide range of cellular stimuli including oestrogen, androgens, insulin, glucocorticoids and serum, during the process of learning and memory or upon metabolic shifting [71, 72, 73, 74, 75, 76, 77]. Remarkably, DNA cleavage by a restriction endonuclease was able to substitute for TOP IIβ in this capacity, suggesting that break formation itself is sufficient for transcription activation [74]. Although breaks generated by DNA topoisomerases are normally transient and easily reversible, the conversion from reversible to irreversible breaks can be accelerated by active transcription [28]. The role of topoisomerases in transcription initiation thereby provides an alternative explanation for why actively transcribed regions exhibit higher rates of break formation.

The second mechanism involves the combined action of TOP I and endogenous endonucleases at RNA‐DNA hybrids. [60, 78, 79] (Fig. 2D). RNA‐DNA hybrids have been shown to induce genome instability by triggering replication–transcription collisions, as discussed above. However, more recent evidence suggests that R‐loops may also induce genome instability independently of DNA replication. In postmitotic cells, endogenous endonucleases can induce SSBs by directly targeting RNA‐DNA hybrids, or single‐stranded DNA exposed by RNA‐DNA hybrids [60, 78, 79]. When these SSBs are in close proximity and on opposing strands, they can result in DSBs upon strand separation. Importantly, exogenous perturbations (i.e. RNA‐DNA hybrid stabilization, TOP I trapping) seem to be necessary to detect these breaks.

Given that R‐loop‐mediated genome instability is typically characterized in cells where RNA‐DNA hybrids are elevated above physiological levels, it remains unclear whether RNA–DNA hybrids are universally toxic in native conditions and how, if at all, they contribute to the break susceptibility observed at actively transcribed regions in physiological conditions. Some insight into this issue comes from two recent studies. Firstly, Promonet *et al*. profiled DSB formation genome‐wide in both unperturbed wild‐type cells and cells with artificially increased RNA‐DNA hybrid levels. In wild‐type cells, endogenous RNA‐DNA hybrids were enriched at both transcription start sites and transcription termination sites, without any corresponding enrichment of DNA DSBs. Conversely, inducing RNA‐DNA hybrids above physiological levels resulted in an accumulation of DNA DSBs at transcription termination sites, which the authors propose reflect an increased incidence of replication–transcription collisions. Therefore, endogenous RNA‐DNA hybrids on their own did not appear to be sufficient to induce detectable DSBs [58]. Secondly, Chakraborty *et al*. [80] reported an enrichment of spontaneous DSBs at RNA‐DNA‐hybrid‐forming sequences in cells exposed to the control solvent DMSO, but not in untreated cells . Together, these studies question the view that physiologically formed RNA‐DNA hybrids induce DNA DSBs, at least at detectable levels, and also emphasize the importance of using appropriate solvent controls when profiling spontaneous DSBs.

### Loop boundary regions: insulators harbour intrinsic fragility

**Fig. 3:**
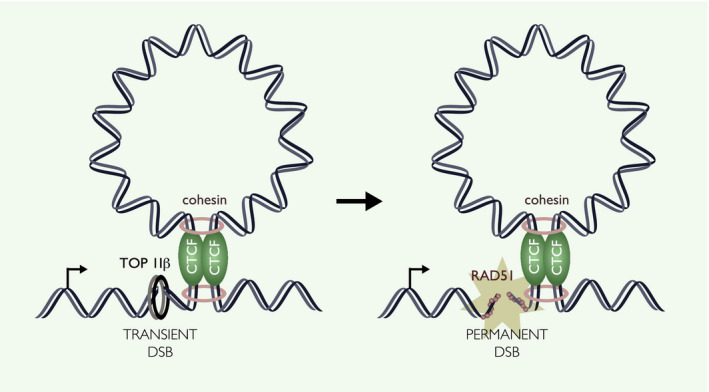
Loop boundaries as sites of genome instability. Boundary regions between TADs are maintained by insulator proteins CTCF and cohesin. TOP IIβ generates transient DSBs to alleviate regional topological stress. TAD boundaries are highly susceptible to DNA breaks even in the absence of exogenous perturbation and especially under conditions of TOP II trapping. The conversion of transient to permanent DSBs is accelerated by nearby transcription. The single‐strand DNA binding protein RAD51 protects against DSBs at loop boundaries.

Another genome instability hotspot identified in these breakomes is the boundary regions between topologically associated domains (TADs). TADs are considered to be the building blocks of the three‐dimensional genome, with an average of ~ 3000 of these insulated domains in mammalian genomes [81, 82, 83]. TADs facilitate key DNA interactions (e.g. between promoters and enhancers), thereby spatially defining regulatory domains and restricting molecular activities within domains [82]. In humans, insulator proteins such as CCCTC‐binding factor (CTCF) and cohesin bind the boundaries of TADs to aid compartmentalization [84, 85, 86]. At TAD boundaries, TOP IIβ also localizes with CTCF and cohesin to resolve any topological constraints imposed by the insulation process [87] (Fig. 3). Recently, a number of breakomes have found that TAD boundaries (i.e. CTCF binding sites) are highly susceptible to breakage in unperturbed conditions and especially when TOP II is trapped with etoposide [10, 26, 27, 28, 29, 30, 88, 89].

Given that topoisomerase‐induced breaks are typically reversible, the molecular mechanisms underlying the susceptibility of TAD boundaries to breakage remain elusive. Early evidence suggests that DSB formation after TOP II trapping is dependent on transcription activity at boundary regions and that depletion of the homologous recombination mediator RAD51 increases break susceptibility at insulator sites [26, 28, 30]. Therefore, it has been suggested that transient breaks induced by TOP IIβ can be turned into persistent DSBs through the act of transcription, requiring the DSB repair protein RAD51 to monitor and repair breaks (Fig. 3). Given the presence of an active repair mechanism, it remains unclear how persistent these breaks would be in healthy cells, although spontaneous breaks are indeed detected at loop boundaries [27, 29, 30]. As an alternative explanation for the DNA breakage frequently observed at loop boundary regions, TAD boundaries are enriched at sites of replication initiation [90, 91, 92]. As discussed below, replication initiation sites are inherently prone to DNA breakage, although the mechanisms underlying this fragility also remain unclear.

### Early‐replicating regions: a poorly understood source of break susceptibility

A surprising outcome of recently profiled breakomes is the identification of early‐replicating regions as being highly susceptible to DNA breakage. Indeed, while there have been sites identified as ‘early‐replicating fragile sites’ (ERFSs) since 2013, it now appears that the susceptibility to breakage extends beyond these originally defined sites to all early‐replicating regions. ERFSs were defined in primary mouse cells as regions enriched with DNA damage marker γH2A.X or bound by DNA repair proteins [RPA and Breast cancer 1 gene (BRCA1)] in response to replication stress [93]. Analogous to CFSs, discussed extensively in the following section, ERFSs are expressed as gaps in metaphase spreads, which were believed to represent DNA breakage. However, while CFSs tend to be found within late‐replicating, AT‐rich regions and within large isolated genes, ERFSs are largely early‐replicating, GC‐rich and within highly transcribed gene clusters. Supporting ERFSs as sites susceptible to breakage, more recent studies have confirmed ERFSs as break site hotspots, or identified similar sites in other organisms [40, 94, 95].

More strikingly, the study that has investigated the susceptibility of ERFSs to DNA breakage find that the vast majority (76%) of DSBs, while being within early‐replicating regions, are outside of formally defined ERFSs [40]. This suggests that early‐replicating regions as a whole may be inherently susceptible to breakage upon replicative stress regardless of their coincidence with the originally defined ERFSs. Although it is perhaps expected that breaks occur at early‐replicating regions under conditions of drug‐induced replication stress, there is some indication that the act of early replication itself predisposes breakage even in unperturbed conditions. For example, studies correlating breakome hotspots with epigenomic features identify early replication timing as the single greatest predictor of DSB formation in native conditions in both cancer and normal cell lines (i.e. Michigan Cancer Foundation‐7 (MCF7) and Normal human epidermal keratinocytes (NHEK), respectively) and using three independent DSB detection assays [89].

**Fig. 4:**
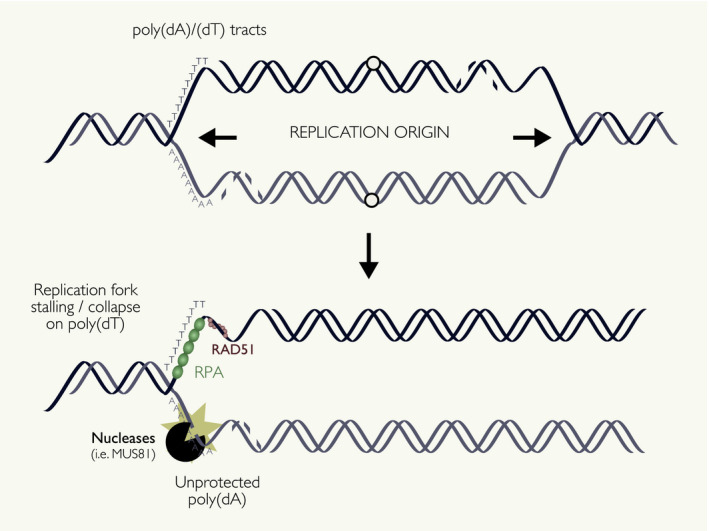
Early‐replicating regions as sources of genome instability. Early‐replicating regions can be susceptible to breakage in the absence of any exogenous perturbation, outperforming markers of active transcription as predictors of DNA break susceptibility. The mechanisms of DNA breakage at early‐replicating regions remain unknown, but it is possibly mediated by poly(dA:dT) tracts. Poly(dA:dT) is conserved repetitive elements, forming intrinsically open chromatin and acting as replication and transcription initiation sites. Poly(dA:dT) tracts are unable to bind protective single‐stranded DNA binding proteins RPA and RAD51, possibly making them susceptible to DNA breakage by endonuclease activity.

Due to the high gene density in early‐replicating regions, the induction of these breaks could be explained by replication–transcription collisions that are more likely to occur in these regions. However, almost none of the break sites within early‐replicating regions occur within gene bodies [40]. In addition, early replication timing was found to be more predictive of DSB formation than the classical marks of active transcription such as polymerase IIβ occupancy or DNase hypersensitivity, suggesting that these breaks are primarily triggered by the events uniquely associated with early replication, independently of transcription [89]. An alternative hypothesis is that a common feature of both early replication and active transcription might be imparting this shared break susceptibility (Fig. 4). Tubbs *et al*. proposed that break susceptibility at early‐replicating regions is due to the presence of poly(dA:dT) tracts present in these regions. Poly(dA:dT) tracts are conserved repetitive elements in eukaryotes that have reduced nucleosome binding, making them form intrinsically open chromatin [96, 97, 98]. Poly(dA:dT) tracts have also been associated with replication initiation sites in lower eukaryotes and transcriptional activation [99, 100, 101]. Therefore, poly(dA:dT) tracts may play critical roles in both replication initiation and transcription control, at the expense of genome stability. Tubbs *et al*. propose that poly‐dA strands are unable to bind the protective single‐stranded DNA binding protein replication protein A (RPA) when unwinding during DNA replication. Poly‐dA strands thereby act as polar replication fork barriers (RFBs), resulting in breaks at replication initiation zones and early‐replicating genes. It is noteworthy that this would occur even in the absence of exogenous replication stress. In line with this notion, the DNA repair factor RAD51 similarly fails to bind the highly rigid poly‐dA tracts, preventing the homology‐directed repair of any breaks that do occur in these regions [102]. Nevertheless, the striking susceptibility of early‐replicating regions to DNA breakage remains to be further investigated.

The observation that early replication, rather than late replication, acts as a better predictor for breakage challenges the widely accepted paradigm that late‐replicating regions are more likely to be genome instability hotspots. This paradigm is conceivably based on the observation that late‐replicating regions tend to form visible gaps in mitotic chromosome following exposure to replication stress [103, 104] and that mutation rates are higher in late‐replicating regions [105, 106]. However, recently published breakome datasets provide evidence that early‐replicating regions may in fact be far more susceptible to DNA breakage in the absence of exogenous perturbations such as drug‐induced replication stress. The discrepancy in mutation rates can potentially be explained by considering the repair pathways and opportunities afforded to either early‐ or late‐replicating regions. For example, early‐replicating regions, while perhaps being more susceptible to breakage, would have more time to complete repair before entry into mitosis. In addition, there is evidence that high‐fidelity homology‐directed repair mechanisms are more frequently detectable in early‐replicating regions [107]. Late‐replicating regions may also experience depleted or imbalanced DNA nucleotide pools which can increase local mutation rates specifically at late‐replicating regions [108]. Together, these may explain why mutation rates appear higher at late‐replicating regions.

### Fragile sites: not so fragile after all?

**Fig. 5:**
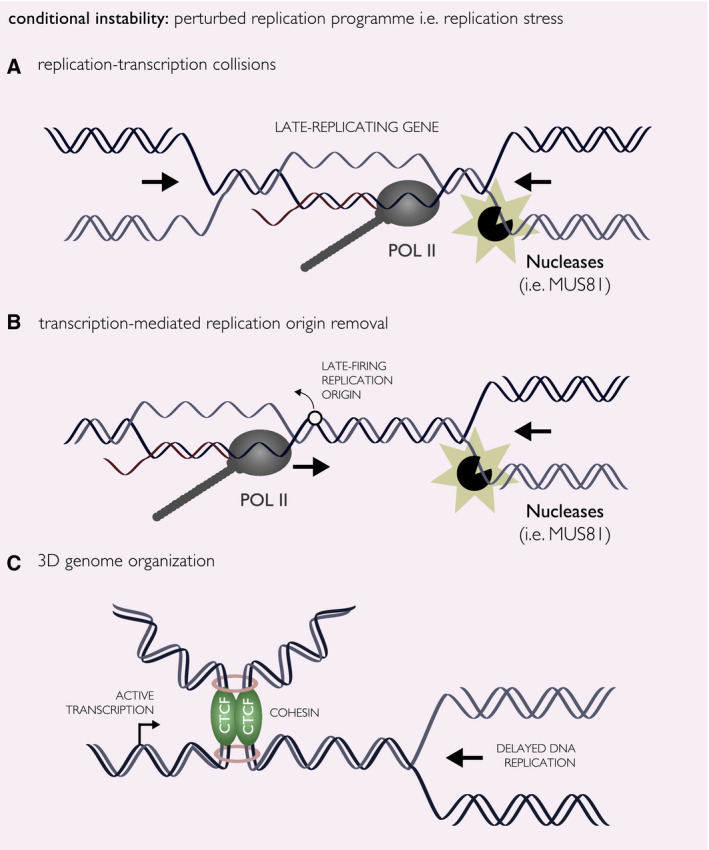
CFSs as sources of genome instability. In response to replication stress conditions, CFSs form visible gaps on metaphase spreads. Three models explaining underlying mechanisms for CFSs formation and associated genome instability are depicted. (A) The canonical model that CFS fragility is mediated through replication–transcription collisions at long genes during late DNA replication. These collisions result in endonuclease‐mediated cleavage of the stalled or collapsed replication fork to initiate replication restart late in DNA replication, or even in mitosis. (B) Transcription of long genes may remove late‐firing replication origins, increasing the distance by which single replication forks have to cover without stalling or collapsing. (C) A more recent model proposing the involvement of 3D genome organization in CFS instability. In this model, delayed replication, active transcription and proximity to TAD boundaries are all required to predict CFS expression in response to replication stress.

Fragile sites are genomic regions originally identified as chromosomal gaps on metaphase spreads and sites of subsequent chromosomal rearrangements, both of which are observed in response to replication stress [13, 109, 110]. Fragile sites are classified as either CFS (found in all human genomes) or rare fragile sites (RFS, found in only 5% of human genomes). CFSs replicate late in the cell cycle and frequently overlap with very long (> 300 kbp) genes [103, 111, 112]. Historically, the mechanism of CFS instability has been proposed to be based on replication–transcription collisions (Fig. 5A), as CFSs typically overlap with large transcriptional units and the formation of cytological gaps at CFSs is dependent on the transcription of these long genes [39, 41]. However, this model has been challenged by more recent studies. For example, it has been demonstrated that transcription at late‐replicating genes removes licensed and unfired replication origins, forcing single replication forks to replicate large stretches of DNA [104, 113, 114] (Fig. 5B). Such events might increase the chance of forming stalled or collapsed replication forks that can then be targeted by endonucleases for cleavage [8, 50], providing an alternative explanation for gap formation. Emerging evidence also proposes an involvement of nuclear architecture in the expression of CFSs. Namely, delayed replication upon replicative stress and transcriptional activity is necessary, but not entirely sufficient, to predict gap formation at CFSs. Instead, gap formation also requires the presence of TAD boundaries at these large, late‐replicating, transcribed genes [115, 116] (Fig. 5C), or the attachment of stressed replication forks to the nuclear matrix [117, 118]. While most of these models involve eventual DNA breakage as the source of CFS instability, it cannot be totally excluded that cytological gaps formed at CFSs actually represent chromatin compaction defects rather than physical DNA breaks. Indeed, recent evidence shows that CFSs have faulty condensin loading following replication stress, which would impede chromatin compaction during mitosis and form the chromosomal gaps visible on metaphase spreads [119].

DNA breakomes, identified by direct DSB detection, could address at least two key questions with regards to CFSs. Firstly, they can determine whether the gaps formed on metaphase chromosomes corresponds to physical DNA breakage or some other form of DNA instability, such as chromatin condensation defects. Three of the published breakome studies, all of which were performed upon exposure to low‐dose replication stress, have commented on an association between DNA break sites and CFSs [23, 40, 80]. Specifically, Crosetto *et al*. [23] were the first to report that many CFSs were susceptible to breakage upon low‐dose replication stress in human cells in 2013. In line with this observation, Tubbs *et al*. in 2018 identified clusters of breaks within the mouse orthologs of human CFS‐associated genes (Fragile site, aphidicolin type, common, fra(16)(q23.2) (FRA16D) and Fragile site, aphidicolin type, common, fra(3)(p14.2) (FRA3B)), albeit that CFSs have not been formally defined in mice. This study also identified a significant enrichment of DNA breaks within poly d(A:dT) sequences of long (> 500 kbp), late‐replicating and highly transcribed genes. DNA breaks within these regions were dependent on their active transcription, analogous to human CFSs [40]. An independent study using indirect DNA break detection methods (i.e. mapping sites of chromosome translocations) has also found evidence of recurrent DSB clusters in certain CFSs upon low‐dose replication stress, suggesting a subset of CFSs undergo physical DNA breakage when exposed to mild replicative stress [21]. Intriguingly, the most recent study, Chakraborty *et al*. in 2020 [80], found no correlation between the DNA break sites identified genome‐wide and CFSs, although the authors speculate that this may be due to differences in solvents. Together, while initial evidence suggests that physical DNA breakage may correlate to CFS gap formation in at least some CFSs, the extent to which metaphase gaps correspond to DNA breakage remains to be fully determined.

Secondly, sequencing‐based breakomes capable of detecting rare events provide opportunities to determine the conditions in which CFSs are susceptible to DNA breakage. As discussed above, CFS gap formation on metaphase spreads is typically observed in cancerous cells or in response to specific replicative stress. It is unclear whether CFS instability (i.e. DNA breakage) is also manifested in unperturbed conditions, but simply below the detection level afforded by cytology‐based assays. This question ultimately addresses whether DNA breakage or instability at CFSs frequently occurs in unperturbed cells, or only when cells have transformed sufficiently to exhibit chronic replication stress. Beyond the three aforementioned studies conducted upon exposure to mild replicative stress, there is a notable absence of CFSs reported as break sites in other studies, even in replicating, highly stressed cancerous cells (Table 1) [120]. This absence ultimately poses questions as to what extent CFSs truly are a source of DNA break susceptibility in physiological settings, despite the longstanding belief that CFSs are frequent hotspots of DNA breakage.

Although chromosomal translocations at CFSs have been observed in cell lines in response to replicative stress *in vitro*, it is now increasingly recognized that CFSs are not frequently sites of gross chromosomal rearrangements in human cancers [21, 121]. This contrasts to well‐described oncogene‐producing chromosomal translocations, for example, 9q34.1 and 22q11.2 resulting Abelson tyrosine‐protein kinase 1 (ABL1)‐Breakpoint cluster region protein (BCR) fusion in Philadelphia chromosome in leukaemia [122]. In addition, CFSs do not overlap with oncogenes. Rather, CFSs appear to overlap with focal deletions observed in cancer cells, including tumour suppressor genes (i.e. Fragile histidine triad protein in FRA3B and WW domain‐containing oxidoreductase in FRA16D) [111, 123, 124, 125, 126, 127, 128, 129]. Nonetheless, the general significance of deletions at CFSs has more recently been called into question, as only one CFS (Xp22.3) deletion was predicted to act as an early somatic driver event among 38 tumour types [130, 131]. Together, these observations support the idea that DNA breakage and rearrangements at CFSs act primarily as secondary events, in response to elevated replication stress, contributing to an already unstable and evolving cancer landscape.

### rDNA, telomeres and centromeres: exploring the fragility of repetitive regions

Given that breakomes have been, to date, based on short‐read sequencing technologies, repetitive regions are typically excluded from analyses due to being difficult to align with read‐mapping algorithms [132]. This is despite the fact that repetitive regions may comprise up to two‐thirds of the human genome and are generally considered to be significant threats to genome stability [133, 134]. However, a few breakome analyses have included repetitive regions, as outlined below. From these studies, it appears that certain repetitive regions can indeed act as hotspots for genome instability, but sometimes only in perturbed conditions such as drug‐induced replicative stress.

#### rDNA

The ribosomal RNA‐encoding genes (rDNA) are composed of long (~ 45 kbp) tandem repeat arrays, which display a high level of transcription activity throughout interphase. Within rDNA arrays, a unique DNA structure that blocks replication fork progression is found at the 3′ end of each transcribed genes. This structure, named RFBs, inhibits the movement of replication machinery in the direction opposite to rDNA transcription, thereby preventing transcription–replication collisions [135]. Nonetheless, with programmed fork stalling at RFBs and high transcriptional activity, rDNA repeats are frequently proposed to be a prominent source of genome instability [136]. Indeed, rDNA loci are hotspots for recombination in cancer cells [137], although whether they are a source of genome instability in physiological settings remains unclear. The notion that rDNA repeats are instability hotspots is supported by a recent report by Zhu *et al*., in which breaks at rDNA RFBs were identified in the budding yeast *S. cerevisiae* in unperturbed conditions. In this study, these breaks were further shown to be specific to cells in S‐phase, but not G1, suggesting that endogenous replication pausing at RFBs can result in spontaneous DSBs within the rDNA locus. In mouse cells, Tubbs *et al*. similarly identified spontaneous DNA DSBs at the rDNA RFBs, once again dependent on active DNA replication. Intriguingly, breakage at RFBs decreased with both low‐ and high‐dose replication stress treatments, suggesting that these breaks are specific to unperturbed replication programmes [40]. To our best knowledge to date, however, enrichment of DNA breaks at rDNA loci has not been reported in human DNA breakomes, and it remains to be elucidated whether spontaneous DNA breakage also occurs at human rDNA repeats.

#### Telomeres

**Fig. 6:**
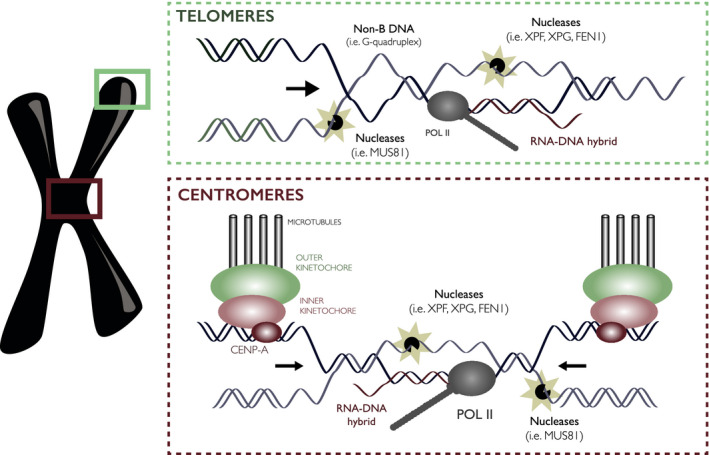
Telomeres and centromeres as genome instability hotspots in response to replication stress. In response to replication stress, DNA breaks can be detected at telomere‐associated repeats and alpha‐satellite repeats, the main constituent of mammalian centromeres. At telomeres, RNA‐DNA hybrids, non‐B DNA structures (loop structures, G‐quadruplexes), and transcription can impede replication fork progression. At centromeres, RNA‐DNA hybrids, non‐B DNA structures, transcription, and high protein occupancy can impede replication fork progression. In both scenarios, persistent RNA‐DNA hybrids or failure to complete replication can provoke DNA breakage by endonucleases (i.e. XPF, XPG, FEN1, MUS81).

Telomeres are composed of very short (6‐bp) repeats of GC‐rich which have telomere‐specific loop structures, active transcription and high RNA‐DNA hybrid densities [138, 139, 140, 141] (Fig. 6). As all of these can theoretically impede replication fork progression and result in fork stalling and collapse, there is a surprising paucity of breaks identified at or near telomeres in recent breakomes. However, it is often unclear whether telomeres are being included in these breakome analyses, as they are composed of repetitive DNA that is typically filtered out during sequencing processing. In addition, because telomeric ends can be considered as DSBs themselves, certain studies actively exclude them from analysis (e.g. Ref. [27]). One exception to this was Crosetto *et al*. 2013, where they assessed the enrichment of various human repeat sequences within break‐susceptible sites in response to replication stress. They find that indeed, telomere‐associated repeats are enriched within break‐susceptible regions, although the enrichment is small compared to other satellite repeats such as centromeric alpha‐satellite repeats [23]. As long‐range sequencing technologies further develop, the fragility of these repetitive sequences will be an important question to address.

#### Centromeres

Centromeres are composed of relatively longer (173‐bp) repeat arrays, but are also typically excluded from breakome analyses. Centromeric repeats were significantly and reproducibly detected in Crosetto *et al*. as enriched for breaks in response to low‐dose replication stress, suggesting that centromeres are prone to breakage in replication stress conditions. DNA SSBs were also notably enriched in human cancer cells at satellite repeats [29], although it was not commented on whether these included centromere‐specific alpha‐satellites. Chakraborty *et al*. also highlighted an apparent enrichment of DSBs at pericentromeric regions, which are similarly composed of satellite repeats as well as transposons and retrotransposons, although potential mechanisms underlying this fragility had not been examined [80]. Therefore, it seems clear that further work has to be done to assess how, if at all, (peri)centromeric regions are subjected to DNA breakage. Indeed, the repetitive nature of centromeric DNA, active transcription, RNA‐DNA hybrid occupancy, late replication, high protein occupancy and substantial mechanical stress during chromosome segregation, poises centromeres as prime candidates for sites of DNA breakage during DNA replication and perhaps even during chromosome segregation in native conditions (Fig. 6) [142, 143, 144, 145, 146, 147]. Aberrant DNA structures resulting from break repair are also frequently observed at centromeres in the absence of exogenous perturbation, and genome instability deriving from centromeres has recently been a topic of keen interest [148, 149, 150, 151].

It is becoming more commonly accepted that breaks at or near pericentromeric or centromeric regions can result in rearrangements and subsequent arm loss [151]. Direct evidence from Taylor *et al*. [152] demonstrates that CRISPR‐induced breaks near centromeric regions can indeed cause chromosome arm loss. This notion is further supported by reports identifying frequent chromosome breaks around centromeric regions in tumours, suggesting that these breaks may contribute to the genome instability observed in cancer cells [153, 154, 155, 156, 157, 158]. Breaks at these regions may further result in isochromosome formation, the most recurrent abnormality in certain cancers and frequently an early somatic driver event in carcinogenesis [131, 159]. It should be noted, however, that centromeres do not meet the classic predictors of break susceptibility (i.e. early replication, high transcriptional activity, strong enhancer activity) according to computational modelling for DSB susceptibility [89]. Nonetheless, a class of genomic regions that show frequent breakage in carcinogenesis, but with low DSB susceptibility by modelling, are characterized by heterochromatic regions composed of repetitive DNA which the authors propose may correspond to repeat‐rich regions near centromeres.

## Conclusion and Future Perspectives

Genome‐wide mapping of DSBs in mammalian cells has previously posed significant challenges due to technical constraints. Epigenetic markers such as γH2A.X have frequently been used as proxies for breaks [160, 161], but this mark can extend up to megabases around DSBs yet be depleted within kilobases directly surrounding the break. In addition, γH2A.X is dependent on H2A.X densities in chromatin, which can further depend on replication timing and transcriptional activity [88, 160, 161, 162, 163, 164]. Attempts to define the sites of DSBs have also been made through other indirect measurements, such as high‐throughput genome‐wide translocation sequencing or translocation‐capture sequencing [31, 32]. It is noteworthy that, while these translocation‐based approaches capture DNA breakage events over a large window of time, translocations are typically generated by error‐prone repair mechanisms, such as nonhomologous end‐joining, fusing two DSB ends in close proximity [165, 166]. As DNA repair pathway choice and the likelihood of translocation are dependent on the local chromatin environment [167], this may lead to a biased distribution of translocation occurrence, and thereby a biased detection of the history of DNA breakages. In contrast, techniques that directly detect physical DNA breaks offer an unbiased, comprehensive genome‐wide mapping of DSB sites with high space–time resolution. These techniques are beginning to shed new light on how DNA instability is manifested across the genome across various physiological conditions.

The DNA breakomes discussed in this review consolidate known hotspots of genome instability (e.g. actively transcribed regions), but also reveal less expected instability hotspots (e.g. boundary regions between TADs, early‐replicating regions). These studies further challenge current paradigms, for example that endogenous DNA breaks are primarily through replication‐based mechanisms in hard‐to‐replicate regions. Ultimately, DNA breakomes are beginning to objectively determine the regions of the mammalian genome most likely to initiate DNA instability in pathologies such as cancer, premature ageing and neurodegenerative disorders.

An important outstanding question is how the spatial distribution of DNA breakage actually correlates to the resulting DNA instability. As discussed above, DNA repair pathway choice is dependent on local chromatin environments, with actively transcribed regions preferring dedicated and high‐fidelity repair mechanisms [167, 168, 169, 170]. In addition, early‐replicating regions may have a higher chance of being repaired before entry into mitosis. Therefore, there may be a discrepancy between DNA DSBs and the mutagenic events that ultimately influence disease onset and progression. Indeed, computational modelling reveals several instances of mismatches between predicted DSBs and structural variant densities observed in tumours [89]. It will therefore be critical to assess genome mutations and rearrangements alongside DNA breakomes to further understand how breakomes shape genome evolution, and vice versa, throughout disease progression.

As the era of next‐ and third‐generation sequencing technologies develop, the detection and quantification of DNA breaks on a genome‐wide scale will be critical to objectively profiling genome instability hotspots in various pathologies, and understanding the mechanisms protecting and repairing breaks in a spatially sensitive manner. Recent technological advancements, for example long‐read sequencing approaches, may accelerate the assessment of the prevalence of DNA breaks in more enigmatic regions of the genome such as repetitive regions (e.g. rDNA, telomeres and centromeres), allowing us to understand how repetitive elements truly contribute to genome instability. In addition, the question of cell‐to‐cell variance in DNA break profiles remains to be resolved. Identifying such variability at single‐cell level may elucidate break prevalence and distribution throughout the population, or whether certain break profiles correspond to cell cycle or transcription status.

These technologies ultimately have the potential to address key questions in the field. Understanding innate sources of vulnerability in the human genome in an unbiased fashion will inform what sorts of exogenous threats are most likely to provoke DNA instability, and thereby initiate and drive human disease. Further, breakomes conducted in specific disease states may identify conditional vulnerabilities which may then guide, for example, the development of safe and effective chemotherapeutics, with minimal off‐target effects to noncancerous cells. Another important outcome of these studies is the observation that DSB profiles vary notably between cell types, and certain features are found to be predictive of DSB incidence only in certain cell lines [30, 89]. This variance in cell responses calls for further investigation into why and how different cell types exhibit different instability hotspots. Given this intrinsic variance in DNA break profiles, other important questions include how DSB profiles vary between human tissues, or change throughout a single‐cell cycle, stem cell differentiation, or cellular and organismal ageing. Addressing these questions will help to delineate how genome instability is invoked and protected against throughout our lifetimes and in human disease.

## Conflict of interest

The authors declare no conflict of interest.

## Author contributions

XS searched the literature and drafted the manuscript. FE critically reviewed and revised the manuscript. XS and FE contributed equally to edit and revise the final version.
